# Immunoescape of HIV-1 in Env-EL9 CD8 + T cell response restricted by HLA-B*14:02 in a Non progressor who lost twenty-seven years of HIV-1 control

**DOI:** 10.1186/s12977-022-00591-7

**Published:** 2022-03-26

**Authors:** Ana Moyano, Oscar Blanch-Lombarte, Laura Tarancon-Diez, Nuria Pedreño-Lopez, Miguel Arenas, Tamara Alvaro, Concepción Casado, Isabel Olivares, Mar Vera, Carmen Rodriguez, Jorge del Romero, Cecilio López-Galíndez, Ezequiel Ruiz-Mateos, Julia G. Prado, María Pernas

**Affiliations:** 1 Virología Molecular, Laboratorio de Referencia e Investigación en Retrovirus, Centro Nacional de Microbiología, Instituto de Salud Carlos III, Carretera de Pozuelo a Majadahonda Km 2, 28220 Madrid, Spain; 2 Max Von Pettenkofer Institute and Gene Center, Virology, National Reference Center for Retroviruses, Faculty of Medicine, LMU München, Munich, Germany; 3 IrsiCaixa AIDS Research Institute, Crta Canyet SN, Badalona, 08916 Barcelona, Spain; 4 Autonomous University of Barcelona, Cerdanyola del Vallès, Barcelona, Spain; 5 Institute of Biomedicine of Seville (IBiS)/Virgen del Rocío University Hospital, CSIC, University of Seville, Seville, Spain; 6 Molecular Immunobiology Laboratory, Immunology Section, Hospital Gregorio Marañón, Madrid, Spain; 7 Department of Biochemistry, Genetics and Immunology, University of Vigo, 36310 Vigo, Spain; 8 CINBIO, University of Vigo, 36310 Vigo, Spain; 9 Galicia Sur Health Research Institute (IIS Galicia Sur), 36310 Vigo, Spain; 10 Centro Sanitario Sandoval. Hospital Clínico San Carlos, IdISSC, Madrid, Spain; 11 Germans Trias I Pujol Research Institute (IGTP), Badalona, Spain

**Keywords:** Long-term non-progressor (LTNP), Loss of viral control (LVC), Env-EL9 escape HLA-B*14:02, CD8 + T-cells

## Abstract

**Background:**

Long-Term Non-Progressors (LTNPs) are untreated Human Immunodeficiency virus type 1 (HIV-1) infected individuals able to control disease progression for prolonged periods. However, the LTNPs status is temporary, as viral load increases followed by decreases in CD4 + T-cell counts. Control of HIV-1 infection in LTNPs viremic controllers, have been associated with effective immunodominant HIV-1 Gag-CD8 + T-cell responses restricted by protective HLA-B alleles. Individuals carrying HLA-B*14:02 control HIV-1 infection is related to an immunodominant Env-CD8 + T-cell response. Limited data are available on the contribution of HLA-B*14:02 CD8 + T -cells in LTNPs.

**Results:**

In this study, we performed a virological and immunological detailed analysis of an HLA-B*14:02 LNTP individual that lost viral control (LVC) 27 years after HIV-1 diagnosis. We analysed viral evolution and immune escape in HLA-B*14:02 restricted CD8 + T -cell epitopes and identified viral evolution at the Env-EL9 epitope selecting the L592R mutation. By IFN-γ ELISpot and immune phenotype, we characterized HLA- B*14:02 HIV-1 CD8 + T cell responses targeting, Gag-DA9 and Env-EL9 epitopes before and after LVC. We observed an immunodominant response against the Env-EL9 epitope and a decreased of the CD8 T + cell response over time with LVC. Loss of Env-EL9 responses was concomitant with selecting K588R + L592R mutations at Env-EL9. Finally, we evaluated the impact of Env-EL9 escape mutations on HIV-1 infectivity and Env protein structure. The K588R + L592R escape variant was directly related to HIV-1 increase replicative capacity and stability of Env at the LVC.

**Conclusions:**

These findings support the contribution of immunodominant Env-EL9 CD8 + T-cell responses and the imposition of immune escape variants with higher replicative capacity associated with LVC in this LNTP. These data highlight the importance of Env-EL9 specific-CD8 + T-cell responses restricted by the HLA-B*14:02 and brings new insights into understanding long-term HIV-1 control mediated by Env mediated CD8 + T-cell responses.

**Supplementary Information:**

The online version contains supplementary material available at 10.1186/s12977-022-00591-7.

## Background

Long-term non-progressors (LTNPs) constitute a small proportion (5–10%) of untreated HIV-1 infected individuals that remain with CD4 + T-cell counts higher than 500 cells/mm^3^. A subset of LTNPs termed viraemic controllers, maintain viral loads between 50 and 2000 HIV-RNA copies/ml [1, 2]. However, a third of these individuals eventually experience an immunological and clinical decline over time [3] and lose their status [4]. Only 1–4% of HIV-infected LNTPs are progression-free survivors after 15 years of follow-up [5].

The immunological and viral factors involved in LTNP status, and how they may contribute to the understanding of spontaneous HIV-1 control, constitute a topic of major interest in the HIV field [2]. While some studies associate long-term control with attenuated viruses [6, 7], others identify non progressor individuals infected with replication-competent viruses [8]. These findings support the idea that immunological factors are also responsible for the LTNPs status. The enrichment of certain HLA class I molecules, including HLA-B*57, -B*27, -B*13, -B*58 or -Cw*3 [9, 10], as well as highly functional HIV-1-specific-CD8 + T-cell responses have been strongly associated with LTNPs. In fact, immunodominant HLA-B*27 and HLA-B*57-restricted CD8 + T-cell responses mediate their effect by targeting highly conserved regions of HIV-1 Gag, which result in a high mutational viral fitness cost [9, 11–13]. On the contrary, in controllers without protective HLA class I alleles, Gag CD8 + T-cell responses do not appear to play a significant role in viral control [13, 14].

Although the presence of the HLA-B*14 allele is common in viraemic individuals [10], the occurrence of HLA-B*14:02 is more frequently associated with viraemic control than HLA-B*14:01 [15]. Particularly, HLA-B*14:02-restricted CD8 + T-cell responses demonstrate immunodominance towards HIV-1 Env [15]. HLA-B*14:02 restricted CD8 + T-cell responses target an epitope located in Gp41, the Env-EL9 [16–18]. Due to the high plasticity of the Env protein, it has been hypothesized that Env-EL9 escape mutations may not confer a significant impact on viral fitness, although some previous studies describe mutations in this epitope related to loss of CD8 + T-cell recognition and lower viral fitness [17, 19].

Here, we identified an HLA-B*14:02 viraemic LTNP that lost spontaneous HIV-1 control after more than 27 years from diagnosis. To study what are the key mechanisms to maintain the non progressor status and what leads to LVC, we performed an in-depth characterization of clinical, virological and immunological factors using biological samples collected over a period of 13 years. The analysis of the viral quasispecies identified the predominance of viral escape variants that target Env-EL9. Additionally, we monitored the magnitude, breadth and function of the Env-EL9 and Gag-DA9 specific-CD8 + T-cell responses in this individual, and determined the impact of the mutational escape in Env-EL9 on the virus infectivity and protein stability.

## Results

### Phylogenetic analysis of HIV-1 *env* in a viraemic LTNP over 13 years.

**Fig. 1:**
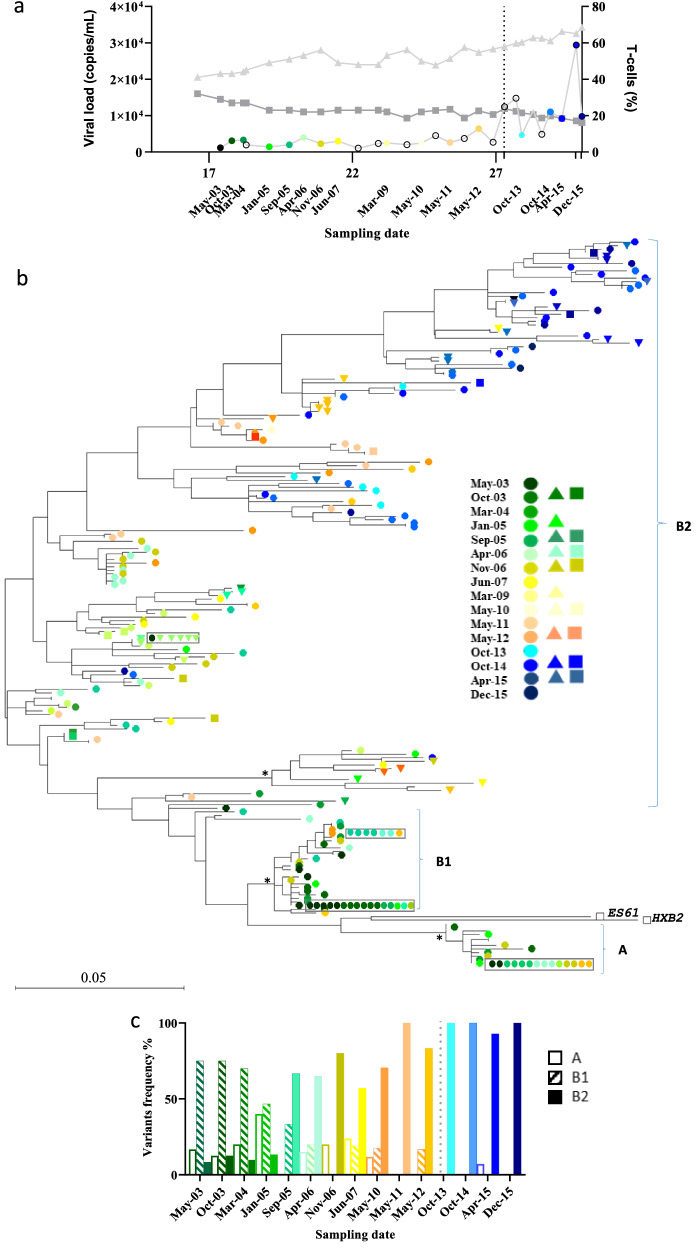
Phylogenetic analysis of HIV-1 *env* evolution during clinical follow-up. **a** Clinical follow-up. Viral load (circles), percentage of CD4 + (squares) and CD8 + T cells (triangles) during the patient follow-up are represented. The x axis refers to years after HIV-1 diagnosis. Colored circles indicate samples taken during the patient follow-up and that are included in the phylogenetic analysis. The dotted grey line separates samples before and after LVC. **b** Phylogenetic analysis revealed the presence of three HIV-1 variants. Two different viruses (A and B) and two different subpopulations (B1 and B2) are represented. A Maximum likelihood (ML) phylogenetic tree based on the C2-V5 *env* region was constructed with 201 unique sequences obtained from proviral DNA (circles), plasma viral RNA (triangles) and virions co-cultured RNA (squares). The sequences obtained at different times points during the patient follow-up are color coded. The identical sequences are included in boxes. Branch lengths are presented as the number of substitutions per site. Bootstrap values above 85% are shown with asterisks. ES61 and HXB2 sequences were included as outgroup viruses to root the phylogenetic tree. c. Replacement of viral populations in proviral sequences during follow-up. A, B1 and B frequency (%) obtained at different times points are represented color coded. The grey line indicates samples collected after LVC

We analysed HIV-1 evolution in a LTNP individual over a period of 13 years (from May 2003 to December 2015). As shown in Fig. 1a, we observed two different phases in the clinical follow-up. From May 2003 to October 2012 (17 to 27 years after HIV-1 diagnosis), the subject remained asymptomatic in the absence of Antiretroviral Treatment (ART) with a mean viral load of 2,821 HIV-RNA copies/ml in plasma, slow decrease on the percentage of CD4 + T-cell counts (slope -0.78% ± 0.14) and an increase in the percentage of CD8 + T-cells (1.14% ± 0.23) per year. We collected 12 biological samples during the 2003–2012 period. In April 2013, the subject lost viral control after 27 years of HIV-1 diagnosis.

**Fig. 2:**
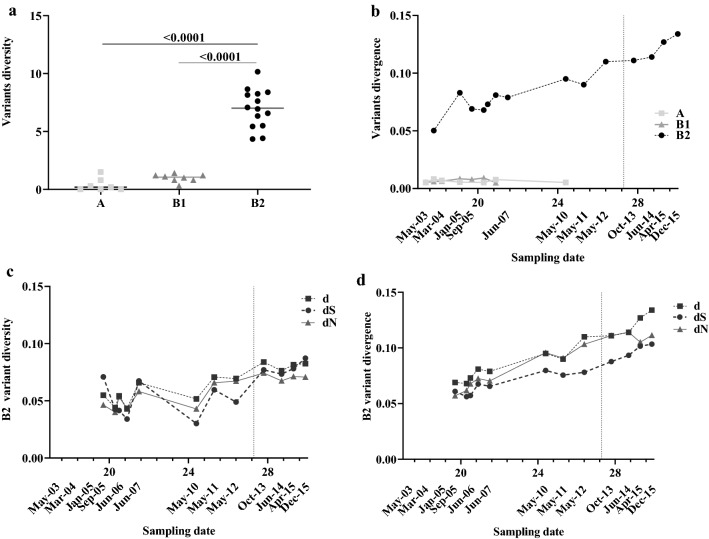
Estimation of viral evolution in the C2-V5 region during the patient follow-up. **a** The C2-V5 *env* diversity (y axis) was calculated as the number of base substitutions per site and averaging from all sequence pairs within each group and for each variant. For statistical evaluations, we used the Tukey's multiple comparisons test considering significance at the 95% (p-values < 0.05 were assumed as statistically significant). **b** The *env* divergence (y axis) was estimated as the pairwise temporal distance from A, B1 and B2 time points (x axis) to the corresponding time to the MRCA. c and d panels show B2 C2-V5 genetic diversity **c** and genetic divergence **d** (y axis) over time (x axis). The plots include the global diversity (squares), number of substitutions per site **d** (circles), number of sites with synonymous substitution per synonymous site (dS) (triangles) and number of sites with nonsynonymous substitutions per nonsynonymous site (dN) (square) from averaging over all sequence pairs between groups

LVC was defined by two consecutive viral loads > 10,000 HIV-1 RNA copies/ml and was identified in four samples during the 2013–2015 period. After April 2013, we observed a 2.48% decrease of CD4 + T-cells and a 2.82% increase of CD8 + T-cells per year. In March 2016, the individual started ART (FTC + RPV + TDF). This LNTP individual was infected by two HIV-1 variants (hereafter referred to as A and B) as previously described by our group [20]. Variant B was segregated into two subgroups, named B1 and B2 (supported by bootstrap values of 97 and 95%, respectively) (Fig. 1b). We extended our previous findings [20] by including new sequences from peripheral blood mononuclear cells (PBMCs), plasma RNA and viral isolates obtained from PBMCs co-cultures during a longer follow-up. The phylogenetic analysis revealed that A and B1 proviral variants were homogenous, with high number of clonal sequences, and were absent from the circulating virus obtained from plasma samples over time (Fig. 1b). By contrast, B2 variant obtained from plasma and viral isolates showed a differential clustering before and after LVC. B2 plasma sequences preceding LVC clustered mostly together, separated from viral isolates and proviral sequences obtained at the same time points. Inversely, B2 sequences after LVC (October 2014, and April, October and December 2015) clustered together independently from their origin (PBMCs, plasma, or isolates). Interestingly, in proviral sequences, while the A variant frequency barely changed over time (mean 12 ± 1.5%), the percentage of B1 changed from 75% in May 2003 to 0% in May 2012 (Fig. 1c). Concomitantly, the B2 variant outgrew B1 by increasing its frequency from 8% (May 2003) to 100% in 2015. We examined the C2-V5 *env* HIV-1 evolution of these three variants. The overall *env* diversity of the B2 sequences was significantly higher than the diversity of A or B1 sequences (Fig. 2a, b). In the C2-V5 *env* region, we found a linear correlation between divergence and sampling date in B2 but not in A or B1 variants (Fig. 2b), indicating a different evolution for each variant. Furthermore, C2-V5 *env* data of B2 showed the number the nonsynonymous (dN) / synonymous substitution (dS) rate ratio > 1, which indicates positive (diversifying) selection in C2-V5 *env* (Fig. 2c–d). These findings demonstrated the B2 variant imposition with extensive evolution in the C2-V5 region.

### Immune characterization of Env-EL9 L592R variants

To investigate the relationship between the acquisition of escape mutations and LVC, we explored individual HLA-restricted epitopes in DNA samples from PBMCs before and after LVC (Table 1). Viral variation mapped mainly in the HLA-B*14:02 restricted epitope Env-EL9, ^584^ERYLRDQQR^592^. We identified two mutations in Env-EL9 from Lys to Arg at position five K588R and Leu to Arg at position nine (L592R). Furthermore, we examined the changes over time, observing the replacement of the Env-EL9 mutant L592R by the double mutant K588R + L592R. In addition, we identified changes associated with putative escape mutations in Gag GY9 and Pol QI9 in the HIV-1 proteome of the B2 variant (Table 1). We detected the Tyr (Y) to Phe (F) mutation at position 9 in GY9 epitope before and after LVC and a secondary mutation at position 5, which never became dominant in the population. Mutation at position 5 from Val (V) to Asp (D) in the QI9 epitope was observed after LVC. Previous work correlated entropy with time to escape [21, 22]. We next calculated Shannon entropy for the different epitopes restricted by the patient HLAs. Of note, the Env-EL9 epitope had a medium entropy value, 0.2093 (medium tercile in our data set) (Table 1) according with previous reports [21].

**Table 1:**
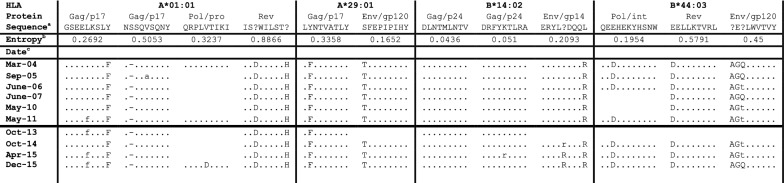
Proviral DNA sequences in patient HLA restricted epitopes from variant B2

^a^Predicted epitope representing the consensus sequence obtained from HIV-1 subtype B. ? denote mix of more than two aa in that position in the HIV data base (https://www.hiv.lanl.gov/content/sequence/QUICK_ALIGNv2/QuickAlign.html)

^b^Shannon entropy values at population level calculated as described in Methods

^c^Date refers to the different time-points sequenced. Samples before and after LVC are separated by a black line

Aa sequence conservation relative to the HXB2 subtype B consensus is indicated with dashes. Lower case letters indicate a mix of two aa, the consensus and the mutated one

**Fig. 3:**
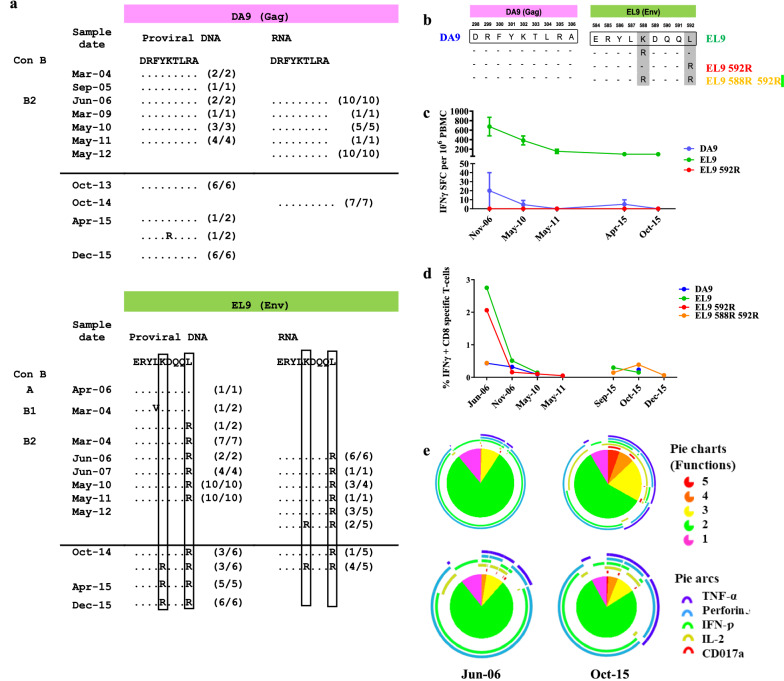
HIV-1 variants in Gag-DA9 and Env-EL9 HLA-B*14:02 restricted epitopes and HIV-specific CD8 + T cell responses. **a** Amino acid (aa) sequence variation in Gag-DA9 and Env-EL9 epitopes from proviral DNA and plasma RNA samples at different time points. Aa sequence conservation relative to the HXB2 sequence is indicated with dashes. The number of observed epitope variants vs the number of sequences analyzed at each time point is shown in parenthesis. A line separates samples collected before and after the LVC. **b** DA9 and Env-EL9 aa sequences representative of the different variants designed to perform the ELISpot and intracellular cytokine staining (ICS). **c** Quantification of HIV-1 specific-CD8 + T cell responses (y axis) against DA9 (blue), Env-EL9 WT (green) and Env-EL9 with the L592R mutation (red), determined by the ELISpot analysis, at different time points (x axis). **d** Gag-DA9, Env-EL9 WT, Env-EL9 with L592R and Env-EL9 592R + 588R specific-CD8 + T-cell responses before and after LVC as the proportion of peptide specific IFN-γ + production by CD8 + T-cells by multiparametric flow cytometry assay. **e** The pie charts show the poly-functionality of Env-EL9 (upper pies) and Env-EL9 with L592R + L588R mutations (bottom pies) specific-CD8 + T-cell responses before (Jun-06) and after (Oct-15) LVC. The pie charts are based on the proportions of cells producing combinations of IFN-γ, TNF-α, IL-2, CD107a and perforin. Single and double production of CD107a and perforin were excluded from the representations

Next, we extended the study to proviral DNA and plasma viral RNA sequences before and after LVC for the mapping of changes at Gag-DA9 and Env-EL9. In proviral DNA, the WT Env-EL9 sequence was detected in A variant while the L592R mutant appeared in 1 out the 2 sequences obtained for the B1 variant (March 04) and in all sequences for the B2 from March 04 to December 15. The second mutation in Env-EL9 (K588R) was detected earlier in plasma samples (May 12) than proviral DNA (October 14). In plasma viral RNA sequences, K558R was a minority (2 out 5 sequences) in May 2012, preceding LVC (Fig. 3a), but became dominant by October 2014 (in 4 out of the 5 sequences). This shift in plasma sequences was also reflected in proviral DNA sequences at the same time. After October 2014, all sequences in B2 displayed the combination of K588R + L592R mutations (Fig. 3a). By contrast, we did not identify amino acid changes in the Gag-DA9 epitope over time (Fig. 3a).

Next, to further investigate the impact of Env-EL9 mutations in CD8 + T-cell immune escape, we synthetized Gag-DA9 and Env-EL9 wild-type (WT) peptides and Env-EL9 peptides containing K558R and L592R variants alone or combined (Fig. 3b). We monitored Env-EL9 restricted CD8 + T-cell responses by IFN-γ ELISpot on PBMCs, and used Gag-DA9-specific CD8 + T-cell responses as a reference. We detected a reduction of Env-EL9 WT responses over time and a lack of responsiveness towards the L592R variant (Fig. 3c). Meanwhile, we did not found any changes and we detected a low frequency of Gag-DA9 responses at all time points. In addition, to further characterize HLA-B*14:02-restricted HIV-1-specific CD8 + T-cell responses, we performed a multi-parametric flow cytometry analysis based on lineage markers and cytokine production. The frequency of functional Gag-DA9 and Env-EL9 specific-CD8 + T-cell responses was monitored by the percentage of IFN-γ + producing cells. Our data showed the decline of IFN-γ + Env-EL9 specific-CD8 + T-cell responses against WT and viral variants over time, presenting a notable decrease between June and November 2006 (Fig. 3d). By contrast, minimal IFN-γ + Gag-DA9 specific CD8 + T-cell responses were found in agreement with the ELISpot data. Additionally, we characterize functional changes of Env-EL9 HLA-B*14:02-restricted HIV-specific CD8 + T-cell response before and after LVC (Oct-15) based on the single or combinatorial expression of TNF-α, perforin, CD107a, IFN-γ and IL-2. We identified cytokine-expressing cells before and after LVC, probably due to an increase of antigen in circulation and viral load upon LVC (Fig. 3e). These results support a stronger immune pressure of HLA-B*14:02 restricted CD8 + T-cells towards the Env-EL9 epitope than to the Gag-DA9 epitope and the selection of CD8 + T-cell HIV-1 escape variants at positions K558R and L592R of the Env-EL9 epitope driven by immune pressure.

### In vitro viral replication of Env-EL9 variants

**Fig. 4:**
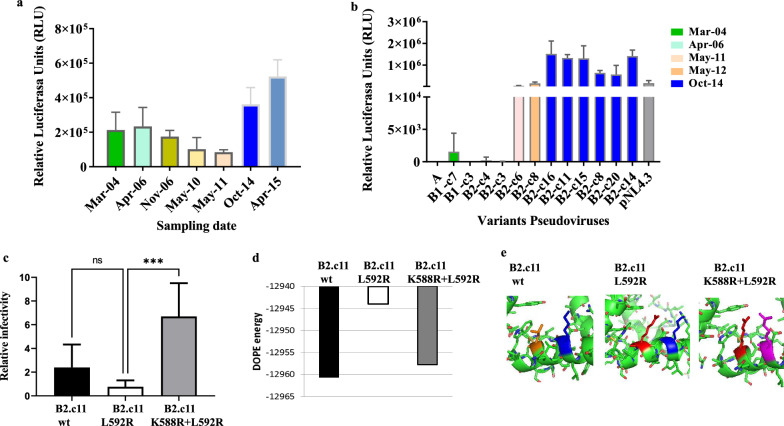
Changes in RC before and after LVC. Infectivity and protein structural stability of EL9 mutants. **a** RC estimated as RLU (relative units of luciferase) production (y axis) of the co-cultured viruses obtained before and after LVC (Oct-14 and Apr-15, blue bars) tested in TZM-bl cells. **b** Infectivity of the pseudoviruses generated with Gp160 Env expression plasmids derived from A (April-06), B1 (c4- May-12 and c7 March- 04) and B2 variants. Four B2 clones from samples previous to LVC (c4, c3, c6 and c8) and 6 B2 clones from Oct-14 (c16, c11, c15, c8, c20, c14) are included. Time points used for pseudoviruses generation was included in brackets. A pNL4-3 pseudovirus is also included as a reference. All these experiments were performed in duplicate **c** We compared infectivity expressed as RLU (relative units of luciferase) for WT, ESC (L592R mutation) and ESC + COM (L592R and K588R mutations) Env-EL9 sequences. Pseudoviruses were constructed from B2 c-11 with L592R mutation by site-directed mutagenesis. The infectivity values were normalized according to the RC of the pNL4-3 control virus (value = 1). Bars represent standard deviation from two experiments with three replicates per experiment **d** DOPE of the protein structure of the WT (black), WT with the escape mutation L592R (ESC, white) and WT with both escape and compensatory mutations (ESC + COM, dark gray). Note that in terms of protein stability, the order is the following: WT (more stable) > L592R with K588R > L592R (less stable). **e** Illustration of the best-fitting 3D structural models (with zoom in the mutation positions) for the WT (the orange and blue positions correspond to those that will suffer the escape and compensatory mutations, respectively), ESC (the red position corresponds to that with the escape mutation) and ESC + COM (the red and pink positions correspond to those with the escape mutation and compensatory mutations, respectively) states

To understand the influence of the HIV-1 replicative capacity (RC) of the different variants over time on the LNTP phenotype, we compared the RC of co-cultured viral isolates obtained before and after LVC. Viruses obtained before LVC showed lower RC than those obtained after LVC (Fig. 4a). Moreover, we next investigated the infectivity of A, B1 and B2 pseudoviruses. We found low infectivity of A and B pseudoviruses (Fig. 4b) while B2 pseudoviruses showed similar levels of infectivity as the pNL4-3 reference virus at the LVC time point (Fig. 4b). These results indicate a potential association between increase of viral isolates and pseudoviruses infectivity and LVC.

Interestingly, L592R variant showed lower infectivity than WT (Fig. 4c). Moreover, the addition of K588R mutation to the L592R mutant resulted in a sixfold (± 1.4) increase compared to the WT in infectivity assays (Fig. 4c). Taken together, these results indicate the compensatory role in infectivity of the K588R mutation.

In order to identify structural changes associated with the L592R and K588 + L592R mutations in Gp41, we evaluated Gp160 protein folding stability by discrete optimized protein energy (DOPE) of the Env-EL9 WT and mutants. Compared to the WT virus, the L592R mutation caused an unstable folding to the overall protein stability. However, the incorporation of the K588R mutation provided higher protein stability (Fig. 4d). A mapping of potential Env-EL9 structural changes showed a close proximity between L592R and K588R mutations, both located in the same α-helix (Fig. 4e), that may explain the compensatory role of the K588R mutation. These results suggest a relationship between the diminished infectivity of the L592R mutant and sustained viral control and the compensatory role of K588R in infectivity and protein stability.

## Discussion

Studies examining the loss of spontaneous control in HIV-1 infected individuals provide essential information to understand the determinants of viral control and pathogenesis. However, longitudinal studies on the loss of LTNP status are limited due to the lack of continuous clinical follow-up [19, 23, 24]. In this study, we overcome some of these limitations by providing long term clinical follow-up, accompanied by the appropriate biological sampling, of an HIV-1-infected individual with LTNP status who lost viral control after 27 years from diagnosis.

In the last years, the number of studies involved in the identification of predictors associated with the loss of LTNP status has increased. Several cross-sectional studies propose HIV-1 DNA load in PBMCs [25], CD4 + T-cell counts after 10 years from seroconversion [4], high viral loads and co-infection with hepatitis C [4], as predictors of loss of LTNP status. Only a few longitudinal studies have been able to directly address the LVC in LTNPs. In previous work, we found that the maintenance of elite controller status was associated with the presence of Gag-specific polyfunctional CD8 + T cell, lack of viral evolution and low levels of pro-inflammatory cytokines [26]. Another study associated the LVC to a combination of viral factors, including X4 co-receptor switching, and loss of antiviral CD8 + T-cell activity [27]. Recently, the impairment of HIV-1 proliferative and cytolytic T-cell effector functions has been observed prior LVC [28].

In this study we have analyzed a dually HIV-infected HLA-B*14:02 LTNP, who lost viral control after 27 years of HIV-1 diagnosis, with the aim of understanding viral and host factors involved in HIV-1 long term control. Our results demonstrate the association between the presence of Env-EL9 immunodominant HLA-B*14:02-restricted CD8 + T-cell responses, viral evolution and immune escape with LVC. In contrast to other studies [29–31], the infection with two heterologous HIV-1 strains was not associated with LVC [20, 24, 32]. Our phylogenetic inferences describe viral evolution [5] and the potential contribution of variant emergence and replacement to LVC. We detected the presence of A and B1 variants in proviral DNA 24 years after HIV-1 diagnosis. Conversely, these variants were absent from plasma and co-culture sources in all time points analysed. According to previous studies, PBMCs contain a pool of recent and archived variants throughout the infection [33], while plasma samples harbour recently produced viral variants and the virions obtained by co-culture generally represent the best fitting variants over time [34]. Based on these previous findings, we postulate that A and B1 variants did not actively replicate over time. This hypothesis is supported by the lack of viral evolution, the absence in plasma or in co-culture samples and the low viral infectivity. It is possible that these variants may be ancient archived variants maintained in proviral reservoirs by homeostatic proliferation events [35] or are defective, which are present in a high proportion in proviral compartments [36]. Consequently, these variants do not seem to have contributed to the LVC in our LTNP case. Therefore, we focused our study on the B2 variant in which the evolution, measured by the genetic diversity and divergence, increased over time.

In this study, we found a continuous shift in viral populations linked to changes in CD8 + T cell responses, as observed between June and November 2006. The reduction in Env-EL9 CD8 + T cell responses paralleled changes in the proportion of A, B1 and B2 variants. The “A” variant sharply decreased around that date (in Apr-06) while escape variant became predominant after this time point. The L592R mutant appeared in one out the two sequences obtained for B1 variant (Mar-04) and in all sequences for the B2 from Mar-04 to Dec-15. Then, the imposition of the L592R mutant in EL9 was concomitant with the changes in CD8 + T-cell responses in 2006.

We also found that spite of the low proportion of WT Env-EL9 variants in proviral DNA sequences, the CD8 + T-cell response targeting WT epitope was detectable over time. The persistence of this response could be explained by a continuous antigenic stimulus, as other proposed [37]. Frater et al. also suggested that this response can maintain selective pressure for HIV-1 escape variants by TCR cross-reactivity [37]. Similarly, we observed the acquisition of the compensatory mutation K588R concomitant with the reduction of WT immune response in May 2011. Additionally, at this point, the CD8 + T-cell poly-functionality increases, probably due to the increase of viral replication and antigen circulation.

HIV-1 evolves by genetic drift as well as by the selection pressure exerted by antibodies and antiviral CD8 + T-cell responses [38, 39]. In LTNPs with protective HLAs class I alleles, CD8 + T-cell responses mostly target highly conserved epitopes in Gag [40–43], which drive the emergence of escape mutants. In the case of the protective HLA-B*14 alleles, present in LTNP particularly for the HLA-B*14:02 allele, CD8 + T-cell responses operate against the Env-EL9 epitope [15]. These data support our findings concerning the immunodominance of Env-EL9 CD8 + T-cell responses over Gag-DA9. Furthermore, this immunodominance of Env responses is reinforced by the identification of escape mutants at this epitope. We focused our study in the immunodominant Env-EL9 epitope restricted by the protective allele HLA-B*14:02 and previously associated with HIV-1 control. In addition, CD8 + T-cell responses restricted by HLA-B are the main driver of HIV-1 evolution and play a crucial role in viral control in contrast to HLA-A haplotypes [9]. Nevertheless, the functional analysis of additional T cells responses would had given us a broader view of other T epitopes involved in LVC. These studies were limited by sample availability. To overcome this limitation, we carried out the analysis of escape mutations in the epitopes restricted by the individual's HLA in the proviral DNA sequences. This compartment reflects all replicating variants throughout the infection [33], so if escape mutations had occurred, such mutations should have accumulated and should have detected in the proviral sequences of PBMC. Beside Env-EL9 mutations, we detected additional changes in Gag-GY9 epitope after LVC. Responses against this epitope restricted by HLA-A*01 has previously described as a subdominant CD8 + T cell responses during the acute phase of HIV-1 infection [44]. Although, we identified changes in the epitope over time, including selecting a double mutant variant before and after the LVC, the new mutant never became dominant. We acknowledge limitations of the analyses restricted to epitopes matched to the patient HLAs and limited depth of sequencing at some time points that may underestimate the presence of additional minority variants.

Patterns of immunodominant T cell responses vary between patients and time. The detailed changes in CD8 + T cell immunodominance in HIV-1 infection can only be monitored from acute infection and with long-term follow-up [21, 22]. During acute infection, immunodominant CD8 + T cells responses target high entropy regions, often Nef and Env where the selection of HIV-1 immune escape is rapid comparing with more conserved epitopes [45]. Conversely, although Env-EL9 entropy is medium, escape mutations in this epitope appear very early in the infection, at least in the context of HLA B*14:02 patients [21]. Our data support the immunodominance of the Env-EL9 CD8 + T cell responses and rapid escape in Env-EL9 by selection of the 592R mutation early in infection, as previously reported [21, 46].

Our results bring novel information, on the impact of Env-EL9 mutations in CD8 + T-cell recognition [15, 17]. In particular, our results on ELISpot and immunophenotyping showed that a non-conservative change of L592R in Env-EL9 conferred the loss of Env-EL9 HIV-1 specific CD8 + T-cell responses. In addition, L592R reduces virus infectivity which it is further compensated by the acquisition of the K588R mutation. Furthermore, the analysis of protein folding stability indicated loss of Env stability by L592R compensated by K588R and according to in vitro observations of infectivity [47]. Troyer et al. found that amino acid changes in Gag usually involve a fitness cost [48]. As a result, those escape variants imposing fitness constrains could contribute to a prolonged control of HIV-1 replication [48]. Likewise, in this study, we conclude that CD8 + T-cell escape mutations in Env-EL9 affect viral infectivity. It is possible that its predominance in the viral quasispecies is related to long-term viral control.

Here, we describe a new HIV-1 escape mutation in Env-EL9 driven by HLA-B*14:02 restricted CD8 + T-cells. We hypothesize that the predominance of L592R immune escape with low infectivity allowed the balance between host immune response and viral replication, contributing to HIV-1 viraemic control for 27 years. When the immunodominant response directed by Env-EL9 declined, the mutation K588R arose in association with a compensatory effect in viral infectivity leading to disease progression.

## Conclusions

Our results reveal the role of Env-specific CD8 + T-cell responses in HLA-B*14:02 LNTP. These results bring new insights into the contribution of non-Gag CD8 + T cell responses in HIV-1 control [15, 45] and support further exploration of Env epitopes and its potential role in HIV-1 spontaneous control.

## Methods

### Study participant

The study participant was an intravenous drug user, HIV-1 subtype B diagnosed in 1986 and without cART treatment during the complete follow-up. The subject carry HLA- A*01:01, A*29:02, and B*14:02, B*44:03 and was clinically followed between May-03 and December-15 (17.4–30 years after the first HIV-1 detection) in an outpatient Clinic Centre (Centro Sanitario Sandoval, Hospital Clínico San Carlos, Madrid, Spain). The individual provided a written informed consent for research purposes of biological samples following the guidelines of the institutional ethical committees of the Institute of Salud Carlos III (CEI PI 05-2010-v3).

### Proviral HIV-1 DNA, plasma and competent-virus RNA isolation

We collected 19 blood samples during the patient’s follow-up and, isolated peripheral blood mononuclear cells (PBMCs) and plasma by the Ficoll-Hypaque gradient. HIV-1 DNA was extracted from PBMCs, using “Speedtools tissue DNA extraction kit” (Biotools B&M Labs, Spain) following the manufacturer’s instructions. Moreover, viral isolates were obtained from PBMCs by co-culturing the patient’s PBMCs with PHA-stimulated PMBCs from three healthy donors in a 1:1 ratio. Co-cultures were maintained in RPMI 1640 (L-glutamine, gentamicin and SFB at 10%) supplemented with IL-2 (10 µg/ml). Every week, 500 µl of the culture supernatants were discarded and fed with 5 × 10^6^ fresh PHA-stimulated donor’s PBMCs. Viruses were harvested when HIV-1 p24 antigen reached > 5000 arbitrary units (25 ng/ml) by electrochemiluminescence immunoassay (Roche Diagnostic, Spain). Viral RNA from plasma samples (n = 11) and viral isolates (n = 6) were extracted using “Nuclisens® aesyMAG ™” (bioMérieux, France) following the manufacturer’s instructions.

### Amplification of HIV-1 *env* C2-V5 region, sequencing and phylogenetic analysis

Proviral DNA and viral RNA were amplified by nested PCR for the *env* gene (C2-V5) by single genome amplification using 169–96 outer and 27–167 inner primers as previously described [26]. DNA was amplified using the Phusion High-Fidelity enzyme (Thermo Fisher, MA, USA). For viral RNA amplification, reverse-transcription-PCR was performed using the One-Step RT-PCR kit (Qiagen, Germany). Next, a total of 1 µl of the first-PCR product was reamplified using the Phusion High-Fidelity enzyme (Thermo Fisher, MA, USA) with the inner primers.

Nucleotide sequences were determined by the Big Dye™ Terminator Cycle Sequencing kit (Applied Biosystems, Thermo Fisher Corporation, MA, USA) in an ABI 3730 sequencer (Applied Biosystems, Thermo Fisher, MA, USA). We used SeqMan Pro v 12.3.1 (DNASTAR) and Bioedit Sequence Alignment Editor (v 7.0.5.3) for assembling and hand-editing [49]. Nucleotide sequences were aligned using MAFFT method (https://www.hiv.lanl.gov/content/sequence/VIRALIGN/viralign.html). Hypermutated sequences identified using Hipermut (https://www.hiv.lanl.gov/content/sequence/HYPERMUT/hypermut.html) were removed prior to phylogenetic inference. Maximum likelihood (ML) phylogenetic tree was inferred with the MEGA program (vX.10) [50] under the General Time Reversible exchangeability matrix, and accounting for variation of the substitution rate among sites and proportion of invariable sites, (GTR + G + I) substitution model which is the substitution model of DNA evolution that best fitted the data. The phylogenetic confidence was assessed by bootstrap analysis using 1,000 replicates. Initial tree(s) for the heuristic search were obtained by applying the Neighbour-Joining (NJ) method to a matrix of pairwise distances estimated with the Maximum Composite Likelihood (MCL) approach. Viral populations were identified by bootstrap values above 85% in the phylogenetic tree and three viral variants (A, B1 and B2) were defined. We calculated the relative percentage of A, B1 and B2 as the number of sequences of each variant at each time point divided by the number of total sequences at that time point.

The mean viral diversity (number of base substitutions per site, by averaging from all sequence pairs within each group) and overall divergence (group mean distance to its respective most recent common ancestor, MRCA) were calculated for the three variants in C2-V5 *env* using the MCL approach. We estimated the number of nonsynonymous substitutions per nonsynonymous site (dN), the number of synonymous substitutions per synonymous site (dS) pairwise distances and dN/dS rate using longitudinal C2-V5 *env* sequences data of the B2 variant. Diversity and divergence estimation for each time point were conducted using the MCL approach. These analyses were conducted using the Kimura 2-parameter (K2P) substitution model of DNA evolution in MEGA vX.10*.*

### HIV-1 amino acid variation in patient HLA restricted-CD8 + T cell epitopes

Twelve optimal epitopes restricted by patient HLA were identified from Los Alamos HIV Database https://www.hiv.lanl.gov/content/immunology/ (Table 1). Amino acid (aa) sequences for the optimal epitopes were obtained from bulk proviral DNA by nested PCR using primers described in Additional file 1 following the conditions described in [51]. Aa changes were studied in longitudinal samples taken before (March-04, Sepember-05, June-06, June-07, May-10, May-11) and after LVC (October-13, October-14, April-15, December-15). Next, we studied viral quasispecies from proviral DNA and viral RNA samples with focus on the immunodominant HLA-B*14:02 restricted epitopes: Gag-DA9 (DRFYKTLRA, aa 298–306) and Env- EL9 (ERYLKDQQL, aa 584–592). To perform this analysis, we carried out a single genome amplification in *gag* and partial *gp41* region by nested PCR using primers described in Additional file 1 following the same PCR conditions described for C2-V5.

Shannon entropy of the 12 optimal epitopes restricted by patient HLA was calculated using Los Alamos database https://www.hiv.lanl.gov/content/sequence/ENTROPY/entropy.html. Subtype B sequence alignment was obtained from https://www.hiv.lanl.gov/content/sequence/QUICK_ALIGNv2/QuickAlign.html.

### Characterization of Gag-DA9 and Env-EL9 HIV-1 specific-CD8 + T cell responses

Based on autologous viral sequences, we designed peptides for Gag-DA9 (^298^DRFYKTLRA^306^), Env-EL9 (^584^ERYLKDQQL^592^) and for the observed Env-EL9 variants at positions L592R (ERYLKDQQ**R**) and K588R + L592R (ERYL**R**DQQ**R**). Peptides were synthetized by EZBiolab (USA) and resuspended following the manufacturer’s instructions. In brief, ELISpot plates (Merck Millipore, MA, USA) were coated with monoclonal antibodies to anti-human IFN-γ (1-D1K, Mabtech, Sweden) overnight at 4 °C. Plates were washed six times with PBS containing 1% FCS. Cryopreserved PBMCs were thawed and 1 × 10^5^ cells/well were cultured in duplicate overnight at 37 °C with 5% of CO_2_ in the presence of Gag-DA9, Env-EL9 or mutated Env-EL9 peptides at 0.01 μg/ml, 0.1 μg/ml, 1 μg/ml and 10 μg/ml. In addition, PHA (250 μg/ml, Sigma-Aldrich, USA) was included as positive control. Finally, plates were revealed with biotinylated anti-human IFN-γ, streptavidin–alkaline phosphatase and its coloured substrate (Mabtech, Sweden). IFN-γ secreting cells were quantified under Immuno Capture and Immuno Spot software to calculate the number of Spot Forming Cells (SFC) as previously described [52, 53]. Wells were considered positive if they contained at least 50 SFCs per 10^6^ PBMCs above the background level (2X mean + 3X standard deviation).

For immunophenotyping**,** cryopreserved PBMCs were thawed, washed and incubated with 10U of DNaseI (Roche Diagnostics, Spain) containing RPMI-1640 (L-glutamine, ampicillin and 10% FCS) medium at 37 °C during 2 h. PBMCs were stimulated at 37 °C with 2 μg/ml of peptides according to sample availability and quasispecies evolution in the presence of 1 μg/ml of CD28/49, 0.7 μg/ml of monensin (BD Biosciences, Spain), 10 μg/ml of brefeldin A (Sigma Chemical Co, USA), and anti-CD107a-BV786 (BD Biosciences, Spain) at the beginning of the incubation. After 6 h of stimulation, cells were surface stained for 30 min with LIVE/DEAD fixable Violet Dead Cell Stain (Life Technologies, CA, USA) for viability, anti-CD14-PB, anti-CD19-PB (Life Technologies), anti-CD56-PB (Biolegend), anti-CD8-PerCP-Cy5.5, anti-CD45RA-FITC and anti-CD27-BV605 (BD Biosciences). Then, cells were washed and permeabilized for 30 min using the cytofix/cytoperm kit (BD Biosciences) and stained intracellularly for 30 min with anti-CD3-APC-H7, anti-IFNγ-PE-Cy7, anti-TNF-A700, anti-IL-2-APC and anti-perforin-PE (BD Biosciences) and then washed twice and fixed in PBS containing 4% paraformaldehyde. Unstimulated condition was included as negative control in each experiment. PBMCs were acquired using a LSR Fortessa Cell Analyzer (BD Biosciences, Spain) and a minimum of 1.5 × 10^6^ total events were recorded for each condition. Lymphocytes were defined as low Forward/Side scatter and expressed CD3 + , and/or no CD8 + , and not CD19, CD14, and CD56.

### Pseudoviruses generation and infectivity assays of Env-EL9 variants

We generated Env-expression plasmid for the different variants. To do this, *gp160* region (HXB2 position aa 2075–2931) was amplified with the primers described in Additional file 1 using Phusion High-Fidelity enzyme following amplification conditions as described [51]. To ensure the presence of variants A, and B1 and B2 throughout the infection, we used several time points for cloning including March 04, April 06, May 11, May 12 and October 14. For variant selection, C2-V5 region from the *gp160 env* sequences were analyzed with a ML phylogenetic tree including representative sequences from the different variants. Amplicons corresponding to A, B1 and B2 variants were purified using the SNAP kit (Invitrogen, MA, USA) and cloned into the pcDNA ™ 3.1 according to the manufacturer’s instructions (Invitrogen, MA, USA). To study the role of the mutations in Env-EL9 epitope, R592L and K588R were introduced into an Env-expression plasmid containing the L592R mutation to generate WT pseudovirus (^584^ERYLKDQQL^592^), and escape + compensatory (ESC + COM) (K588R + L592R). Mutations were introduced by site directed mutagenesis using Quick Change Lightening Site-Directed Mutagenesis Kit (Agilent Technologies, CA, USA) with the primers described in Additional file 1. All mutations were verified by plasmid sequencing. Pseudoviruses were obtained by 293T co-transfection of the Env-expression plasmid in combination with an Env defective HIV-1 backbone by calcium chloride protocol [54]. After 72 h, the pseudoviruses generation was quantified by measuring HIV-1 p24 antigen production in the supernatants by electrochemiluminiscence Immunoassay (Roche Diagnostic).

In addition, we studied viruses RC derived from the co-culture along the following time points: May-04, April-06, November-06, May-10, May-11, October-14 and April-15. To this end, we evaluated RC in 10^5^ TZM cells with 30 units of p24 antigen. TZM-bl cells (NIH-ARP Cat# 8129-442, RRID:CVCL_B478) were cultured in DMEM supplemented with 1% fetal bovine serum cultured and 20 μg/ml DEAE-dextran was added to each well. Three replicates of each virus were included in each assay. After 48 h, we lysed the cells and the production of luciferase was measured “Luciferasa assay system” (Promega). All the assays were performed in duplicated. Pseudoviruses and Env-EL9 mutant’s infectivity was calculated following the same procedure.

### Evaluation of the stability of Gp160 variants

We evaluated the Gp160 stability for the WT amino acidic sequence, the sequence with the escape mutation L592R (ESC variant) and, the sequence with both the escape L592R and compensatory K588R mutations (ESC + COM variant). To do so, first we performed homology modelling with Swiss-Model [55] to obtain the best structural representation (structural model) for the WT, ESC and ESC + COM protein sequences. Next, we computed the protein stability for each previously selected structural model with the discrete optimized protein energy (DOPE); a traditional metric of protein folding stability [56] that is implemented in the well-established framework Modeller [57] The DOPE estimates from every variant (WT, ESC and ESC + COM) can be compared (i.e., [58]) to evaluate the influence of every mutation on the Gp160 stability.

### Statistical analysis

A linear regression analysis for clinical and quasispecies evolution over time was performed with GraphPad Prism (v8.01). For *env* nucleotide divergence and diversity analyses, we applied the Tukey's multiple comparisons test. Differences in viral RC were evaluated with the unpaired t-test. For all the analyses we considered significance at the 95% (p-values < 0.05). We constructed poly-functionality pie charts using Pestle (v1.6.2) and SPICE (v5.2).

## Supplementary Information

**Additional file 1.** Oligonucleotides used for amplification. Table containing all the oligonucleotides used for the different PCR assays described in the Methods.

## Data Availability

Nucleotide sequences for C2-V5 *env*, *gp160* and *gag* sequences were deposited in GenBank with the following accession numbers: MT 530457-530692, MT 530693–530743, MT 530744–530774.

## Abbreviations

LTNPs: Long-Term Non-Progressors
HIV-1: Human immunodeficiency virus type 1
LVC: Loss viral control
ART: Antiretroviral Treatment
PBMCs: Peripheral blood mononuclear cells
MCL: Maximum Composite Likelihood
dN: Nonsynonymous
dS: Synonymous substitution
WT: Wild type
ESC: Escape
ESC+COM: Escape+ compensatory
DOPE: Discrete optimized protein energy
