# Supplementary material for: Immunoescape of HIV-1 in Env-EL9 CD8 + T cell response restricted by HLA-B*14:02 in a Non progressor who lost twenty-seven years of HIV-1 control
Source: Retrovirology. 2022 Mar 26;19:6. doi: 10.1186/s12977-022-00591-7 (PMC8962528; doi:10.1186/s12977-022-00591-7)
Supplement: Supplementary file 1 — Additional file 1. Oligonucleotides used for amplification. Table containing all the oligonucleotides used for the different PCR assays described in the Methods. [file 12977_2022_591_MOESM1_ESM.pdf]

Oligonucleotides used for amplification

	1st PCR primer				2nd PCR primer		
	Fragment	Name	Sequence	Location	Name	Sequence	Location
**HLA epitopes**	**5`LTR-GAG**	**187U**	CACACACAAGGCTACTTCCCT	57-77	**554**	GTCTCTCTTGTTAGACCAGATCTGAGCCTG	456-485
		**147D**	TCTTCTGTCAATGGCCATTGTTTAAC	2610-2635	**352**	CAGTTCCTTGTCGCTCAGCTCCTGCTTC	2211-2238
	**Pol**	**136U**	AAGAGAGCTTCAGGTTTGGGG	2168-2188	**351**	GAAGCAGGAGCTGAGCGACAAGGAACTG	2211-2238
		**33D**	CAAGAAATGGAGCCAGTAGA	5825-5844	**552**	GCAGTTGTTGCAGAATTCTTATTATGGCTTCC	5729-5760
	**gp160**	**294U**	ATGGCTTAGGGCAACATATCTATG	5677-5700	**551**	GGAAGCCATAATAAGAATTCTGCAACAACTGC	5729-5760
		**163D**	CTGAGGGATCTCTAGTTACCAGAG	9664-9687	**555**	CCAGAGTCACACAACCGCGGGGCACACACTACT	9637-9669
**QS analysis**	**gp120**	**169U**	AATGTCAGCACAGTACAATGTACAC	6945-6969	**27**	ATAAGCTTGCAGTCTAGCAGAAGAAGA	7004-7030
		**96D**	AGACAATAATTGTCTGGCCTGTACCGT	7836-7862	**167**	TTCTCCAATTGTCCCTCATATCTCCTCCTCCA	7634-7665
	Gag **DA9**	**505U**	CGAGGGGCGGCGACTGGT	728-745	**171**	TTTGACTAGCGGAGGCTAG	761-779
		**40D**	TTCCCTAAAAATTAGCCTGTCT	2074-2096	**336**	TTCCAACAGCCCTTTTTCCTAGGGG	2009-2033
	Env **EL9**	**570U**	TTGAACCAYTAGGAGTAGCACCCAC	7696-7720	**572**	ACCAAGGCAAAGAGAAGAGTGGTG	7719-7742
		**168D**	AATGGTGAGTATCCCTGCCTAACTCTATT	8340-8368	**282**	TCCTACTATCATTATGAATATTTTTATATA	8265-8294
**Pseudovirus generation**		**176U**	TTCTCCAATTGTCCCTCATATCTCCTCCTCCA	7634-7665	**577**	CACCTAGGCATCTCCTATGGCAGGAAGAAG	5954-5983
		**166D**	TGGAGCCAGTAGATCCTAGACTAGAGCCCT	5832-5861	**576**	GTCTCGAGATGCTGCTCCCACC-	8883-8904
**EL9 mutants**		**R592LU**	TAAAGGATCAGCAG**CTC**CTGGGGATTTGGGG	7984-8014			
	**EL9** WT	**R592LD**	CCCCAAATCCCCAG**GAG**CTGCTGATCCTTTA	7984-8014			
		**K588RU**	CCTAGCTGTGGAAAGATACCTA**AGG**GATCAGCAGCT	7694-7999			
	**EL9** WT+COM	**K588RD**	AGCTGCTGATC**CCT**TAGGTATCTTTCCACAGCTAGG	7694-7999			
		**K588RU**	GTGGAAAGATACTAA**AGG**ATCACAGCGCCTGGG	7971-8005			
	**EL9** ESC+COM	**K588RD**	CCCAGGCGCTGCTGA**TCC**TTTAGGTATCTTCCAC	7971-8005			
